# Static and Dynamic Mechanical Properties of 3D Printed ABS as a Function of Raster Angle

**DOI:** 10.3390/ma13020297

**Published:** 2020-01-09

**Authors:** Mateusz Galeja, Aleksander Hejna, Paulina Kosmela, Arkadiusz Kulawik

**Affiliations:** 1 Department of Material Engineering, Central Mining Institute, Pl. Gwarków 1, 40-166 Katowice, Poland; mgaleja@gig.eu (M.G.); akulawik@gig.eu (A.K.); 2 Department of Polymer Technology, Gdańsk University of Technology, Narutowicza 11/12, 80-233 Gdańsk, Poland; paulina.kosmela@pg.edu.pl

**Keywords:** 3D printing, infill angle, mechanical performance, acrylonitrile butadiene styrene, impact strength

## Abstract

Due to the rapid growth of 3D printing popularity, including fused deposition modeling (FDM), as one of the most common technologies, the proper understanding of the process and influence of its parameters on resulting products is crucial for its development. One of the most crucial parameters of FDM printing is the raster angle and mutual arrangement of the following filament layers. Presented research work aims to evaluate different raster angles (45°, 55°, 55’°, 60° and 90°) on the static, as well as rarely investigated, dynamic mechanical properties of 3D printed acrylonitrile butadiene styrene (ABS) materials. Configuration named 55’° was based on the optimal winding angle in filament-wound pipes, which provides them exceptional mechanical performance and durability. Also in the case of 3D printed samples, it resulted in the best impact strength, comparing to other raster angles, despite relatively weaker tensile performance. Interestingly, all 3D printed samples showed surprisingly high values of impact strength considering their calculated brittleness, which provides new insights into understanding the mechanical performance of 3D printed structures. Simultaneously, it proves that, despite extensive research works related to FDM technology, there is still a lot of investigation required for a proper understanding of this process.

## 1. Introduction

Three-dimensional (3D) prototyping belongs to the most popular topics that have emerged recently. The technology of additive manufacturing and rapid prototyping enabled localization and personalization of production, offering new opportunities to produce small objects at a lower cost with properties difficult to obtain with former technologies, such as injection molding, turning, or milling [1]. Today, the main technologies used in 3D printing are stereolithography (SLA), selective laser sintering (SLS), laminated object manufacturing (LOM), and fused deposition modeling (FDM) [2].

From all 3D printing technologies mentioned above, the most accessible to the public is the FDM technique. FDM is a technology based on additive manufacturing where the plastic filament is being drawn to the extrusion nozzle by the knurled feeder. It allows controlling the material’s flow on demand. Printing nozzle at specified temperature starts to melt and extrude small beads of provided thermoplastic material, simultaneously being moved at horizontal and vertical directions, which results in a layer-by-layer deposition. Each layer can be considered as a sliced horizontal cross-section of the final object. The process results in ready-to-use products, as the material hardens after the nozzle is being moved to another layer of the final product [3]. The described approach allows printing objects of any desired shape following computer-aided design (CAD) [4].

In comparison to conventional manufacturing processes used for thermoplastic materials, FDM printing creates near-net-shape components with over 90% material usage. Therefore, it can be considered an environmentally friendly process. Thanks to the use of this technology, production does not require expensive molds to create complex parts [5]. The traditional industrial approach to machining uses subtraction processing guided by computer numerical control (CNC). The main problem starts if the product is too complex for the subtraction processes (cannot meet the specifications and objectives of the product). Over time, the only solution was to increase the precision and complexity of technology. Conversely, additive manufacturing has advantages that subtraction processing lacks [6].

Increased demand for the use of additive manufacturing, as a tool to create functional end-use products, requires a better understanding of the FDM process [7]. Good understanding of various printing parameters and their impact on the structure of resulting products is a necessary step for improving their mechanical properties. Such an approach would enable the implementation of 3D printing into industrial practice at a wider scale and partial substitution of injection molding in applications, in which it is generating high production costs, simultaneously increasing the price of obtained products.

**Figure 1:**
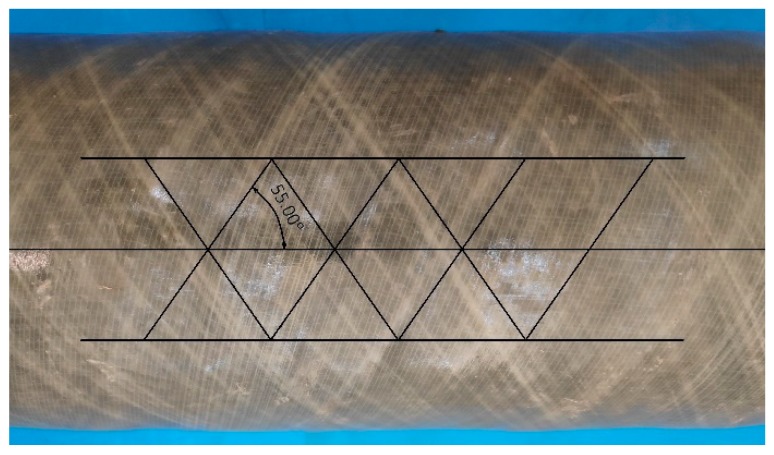
Alignment of fibers in filament-wound pipes.

The structure of FDM printed objects consists of layers behaving similarly to laminate structures, which allows using laminate theories in the mechanical behavior of the parts [8]. Therefore, some insights related to the mechanical properties of materials could be transferred from other materials to 3D printing. A very interesting example is the case of filament-wound pipes. During their manufacturing, rotation angle at each side of the mandrel controls filament winding structure based on the winding angle. The resulting pattern can be presented by a regular diamond-shaped cell with a simple laminated +φ/−φ fiber orientation [9]. In the case of these materials, a winding angle of 55° (see Figure 1) provides the best mechanical performance, especially considering ring stiffness of the material, very important from a technological point of view [10,11].

The main goal of the presented research work is to analyze the influence of fill angle on tensile properties of 3D printed samples, as well as noticeably less frequently analyzed, impact performance and dynamic mechanical behavior to optimize further manufacturing processes [12]. Except for infill configurations conventionally analyzed in other research works, we aimed to investigate the configuration applied in the manufacturing of filament-wound pipes, which provides them the exceptional mechanical properties [13]. In most of the previous works, which investigate a similar topic of the raster angle, specimens were printed only in one direction without corresponding interlayer orientated in the right angle to the previous one, hence layers were deposited perpendicularly [14,15]. Noticeably fewer research works were related to investigations of the varying angle between alternating layers [16], and, to the best of our knowledge, no work has been published, in which 55’° configuration was analyzed, which have been repeatedly proven to provide exceptional properties in the case of filament-wound pipes [17,18]. Moreover, obtained materials were compared to injection molded specimens, as potentially 3D printing aims to replace injection molding, which requires noticeably more complex and more expensive machines.

## 2. Materials and Methods

### 2.1. Materials

Materials used for this study were commercial three types of acrylonitrile butadiene styrene filament differ in properties: ABS-X (black and white) named as 1B and 1W, ABS AT (natural white) named as 2W provided by F3D Finnotech Sp. z o.o. (Katowice, Poland). According to the producer, ABS AT is dedicated for industrial and professional applications, it is characterized with higher thermal stability, and, therefore, it can be processed and work continuously at higher temperatures. ABS AT shows lower value of linear contraction, below 0.4%, while, for ABS-X, it may reach 0.7%. Moreover, comparing to ABS-X, it shows lower emissions of volatiles and absorption of moisture.

### 2.2. Design

Geometric models of test specimens necessary to perform tests have been made according to ISO 527-2-1A (Overall length = 170 mm) and ISO 179-1 (80 × 10 × 4 mm, unnotched Charpy) standards with AUTODESK Inventor software (Inventor: 2018.3 Build: 284, Autodesk, Inc., San Rafael, CA, USA). Preparation of samples for 3D printing manufacturing purposes required making small corrections in specimen models by increasing thickness by 0.15 mm, which corresponds with the thickness of the first layer used to attach the print to the printing table.

### 2.3. Preparation of Samples

Blixet B50-multi FDM 3D printer (Blixet Sp. z o.o., Sosnowiec, Poland) with build size 400 × 400 × 520 mm was used to print models from STL files. GCODE was generated by Repetier Host with Slic3r slicing software (1.3.1-dev, Open-source platform). The design of experiment (DOE) for printer parameters are presented in Table 1 along with fill angles of each specimen.

**Figure 2:**
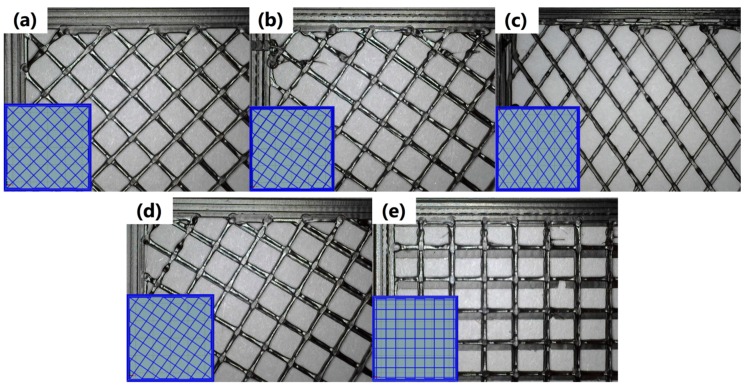
Scheme of layers’ deposition during 3D printing of samples (at 20% infill percentage) for following infill angles: (**a**) 45°, (**b**) 55°, (**c**) 55’°, (**d**) 60° and (**e**) 90°.

All test samples were printed at *XY* layer orientation, specimens GCODE for 45–55–60–90 degree fill angles were created in Slic3r software (1.3.1-dev, Open-source platform) with a standardized translation vector of 90° between combined layers. The detailed scheme of layers’ deposition in each variant is presented in Figure 2.

The only exceptions were samples obtained for 55’° infill angle, which were redesigned in comparison to the original 55° arrangement. In redesigned specimens, layers were deposited at 55° and −55° to the longitudinal direction, which resulted in an increase of the angle between the following layers from 90° to 110°. As mentioned above, this configuration of infill was applied based on the manufacturing of filament-wound pipes. Analysis of the “conventional” 55° raster angle configuration was aimed to compare the influence of mutual arrangement of the following layers on the mechanical performance of obtained samples and to compare a “conventional” approach to the innovative one, based on filament-wound pipes.

Injection-molded samples were obtained using the Arburg type Allrounder 270-210-500 injection-molding machine (Arburg, Loßburg, Germany). Specimens were prepared according to ASTM 527 standard, with the cross-section of the measurement part equal 40 mm^2^. The machine is equipped with a Priamus injection process controller (Priamus, Schaffhausen, Switzerland). ARBURG Allrounder characteristics: screw diameter: 25 mm, injection pressure: max 1400 bar, clamping force: 500 kN. Sample injection parameters: temperature of the polymer melt: 230 °C ± 2 °C, form temperature: −40 °C ± 1 °C, injection speed: 190 mm/s, cycle period: 60 s, injection pressure: 500 bar, clamping pressure: 350 bar.

### 2.4. Measurements

The tensile strength, elongation at break, and elastic modulus were estimated following the PN-EN ISO 527 standard. It is one of the most common mechanical tests performed on a dogbone or dumbbell specimen. The geometry of the test sample allows for a wide section to be easily gripped while the elongation testing and measuring the load carried by a specimen is performed. Collected data about load and deflection allows generating a stress–strain curve which can be used to extract a variety of tensile properties [19]. Tensile tests were performed using the Instron 4465H 1937 tensile testing machine (Instron; Norwood, CO, USA) with elongation head and an extensometer. Tensile tests were performed at a constant speed of 1 mm/min (for Young’s modulus) and 50 mm/min (tensile strength and elongation at break). Five samples were analyzed for each specimen.

The Charpy impact test has been conducted in accordance with PN-EN ISO 179-1:2010 standard. The test has been performed on JSP Maskinfabrik A/S Hammel Pendulum 12959 (SCITEQ A/S; Hinnerup, Denmark) with a pendulum of potential energy 7.5 J. During testing, pendulum is raised to a measured point and after releasing gains speed as it swings toward a mounted specimen of test plastic. At the moment of their contact pendulum loses energy to break the plastic specimen. The lost energy is equated with the energy absorbed by the test sample during the breaking process [19].

The dynamic mechanical analysis was performed using the DMA Q800 TA Instruments apparatus (TA Instruments; New Castle, DE, USA). Samples cut to the dimensions of 40 × 10 × 2 mm were loaded with a variable sinusoidal deformation force in the single cantilever bending mode at the frequency of 1 Hz under the temperature rising rate of 4 °C/min within the temperature range between 20 and 180 °C.

Results of the static and dynamic mechanical analysis were used to calculate the brittleness of investigated materials, according to the following Equation (1) presented by Brostow et al. [20]:

*B* = 1/(*ε_b_* × *E*′) (1)

where: *B*—brittleness, 10^10^ %·Pa; *ε_b_*—elongation at break, %; *E*′—storage modulus at 25 °C, MPa.

## 3. Results and Discussion

**Figure 3:**
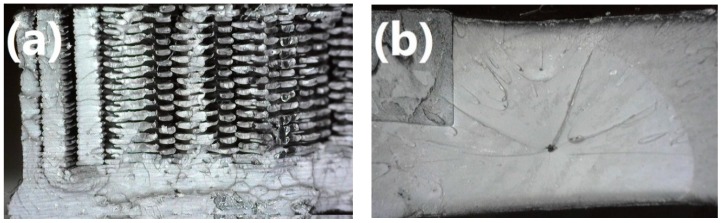
Fracture areas obtained after tensile tests for (**a**) 3D printed sample with infill angle of 55’° and (**b**) injection molded sample.

In Table 2, there are presented values of mechanical properties of 3D printed samples and compared to those of injection-molded materials. As can be noticed, injection-molded samples are characterized with significantly improved mechanical performance comparing to 3D printed ones. Such an effect is related to the structure of the obtained materials. In Figure 3, fracture areas obtained after tensile tests are presented for specimens prepared by both methods. It can be seen that injection-molded samples show homogenous, concise structure, in contrast to the 3D printed specimens. It is related to the characteristics of the process, especially to high pressure applied during the forming of material, which guarantees precise filling of a mold cavity with polymer melt [21]. For better understanding, in Table 2, there are also presented values of materials’ porosity, which were determined according to the following Equation (2):

*P* = (*ρ_inj_* − *ρ_3D_*)/*ρ_inj_* × 100% (2)

where: *P*—porosity, %; *ρ_inj_*—density of the injection-molded sample, g/cm^3^; *ρ_3D_*—density of the 3D printed sample, g/cm^3^.

It can be seen that 3D printed samples are characterized with significantly higher porosity, implicating differences in density, which has a very significant influence on tensile strength of material [22].

Moreover, due to differences in the alignment pattern of filament during 3D printing of specimens, hence different porosity, the orientation of infill has a significant impact on the mechanical performance of the material. Generally, very similar mechanical performance and its dependence on infill angle in the case of 3D printed ABS samples were noted by other researchers [23].

3D printed materials showed the best tensile properties for default 45° infill angle, accounting for 94.5%, 85.3% and 85.3% of the strength of injection molded samples, for 1W, 2W, and 1B filaments, respectively. It may be because specimens with a 45° angle have alternately stacked layers with the optimal orientation to loading direction in the specimen for tension tests. Results differed in 55’° sample case, where overall properties were noticeably (except 2W specimen) worse in a tensile test. Such poor tensile performance was caused by the highest porosity of samples for this infill configuration.

Elongation at break is decreasing with the increase of infill angle, which is related to the mechanisms occurring during tension on the molecular level. As the polymer sample is subjected to uniaxial tension, macromolecular chains are rearranging and aligning in the direction of tension. In the ideal situation and appropriately low strain rate, chains are finally reaching their maximum length before the break. In reality, such a situation is impossible due to entanglements and branching of polymer chains. Nevertheless, when chains are originally aligned closer to the direction of tension, they require lower strain to reach their maximum length, which affects the value of elongation at break. On the other hand, when polymer macromolecular chains are aligned perpendicularly to the direction of tension, they are often unable to rearrange. In such a situation, when adhesion between particular chains is significantly weaker than the strength of the chain itself, delamination may occur [24]. It is especially likely for a 90° infill angle when every second layer is deposited perpendicularly to the direction of strain applied during tensile tests.

**Figure 4:**
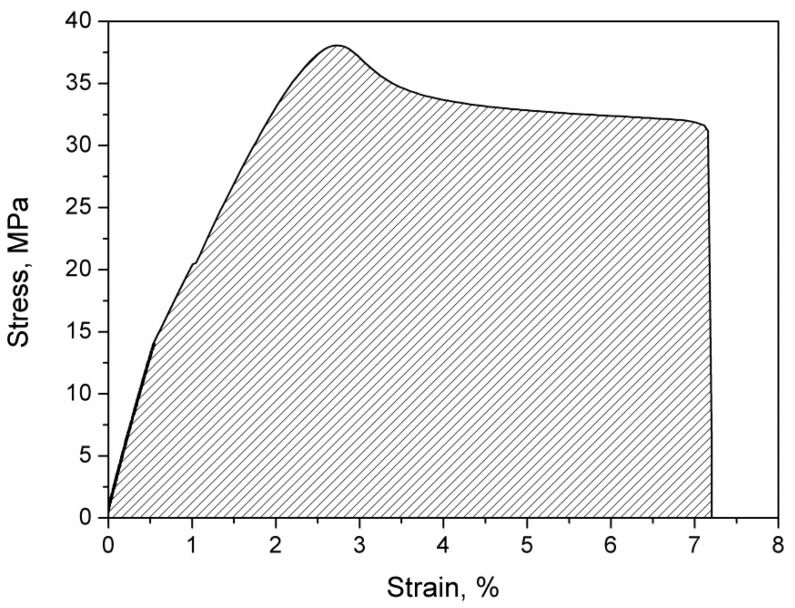
Exemplary stress–strain curve for the 1W45 sample.

In Figure 4, an exemplary stress–strain curve for 1W45 sample visualizing method of calculating toughness by integrating the stress–strain curve. As a combination of material strength and elongation at break, toughness is a measure of the energy that can be absorbed or dispersed by the material before it cracks. The values of toughness for 3D printed samples are presented in Table 2. Ideally, tough material should be able to withstand high stress for possibly the highest elongation. It can be seen that the best combination of strength and ductility was observed for samples with a 45° infill angle. The lowest toughness values were observed for a 90° fill angle, which can be associated with the very low strength of every second layer, in which infill was deposited perpendicularly to the tension direction and low elongation of fibers parallel to tension direction. In general, the drop of toughness with the increase of infill angle can be observed, which is strictly associated with the decrease of elongation at break.

**Figure 5:**
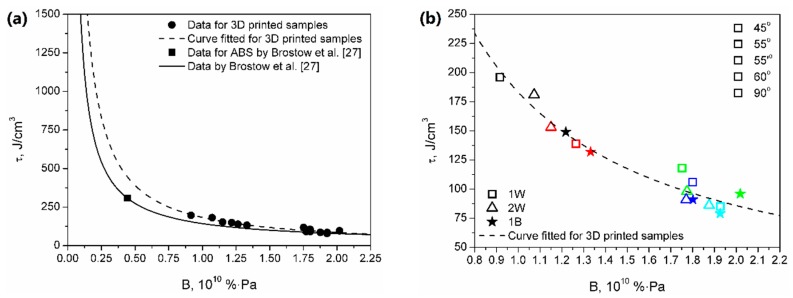
Relationship between toughness and brittleness for analyzed materials. (**a**) Comparison with literature data by Brostow et al. (26), (**b**) Detailed data for 3D printed samples.

Figure 5 shows the dependence between toughness and brittleness of prepared samples. As mentioned above, brittleness was calculated from the results of static and dynamic mechanical tests following Equation (1) proposed by Brostow et al. [20]. Brittleness was determined as a combination of storage modulus and elongation at break. Materials characterized by a low value of this parameter need to be able to withstand possibly high stress at the possibly widest range of strains. Therefore, low brittleness requires simultaneously high modulus and high elongation at break, posing it as an antagonist of toughness. The significance of the relationship between elongation at break and brittleness was earlier emphasized by other researchers [25,26]. Brostow et al. [27] presented the Equation (3), which can be used to mathematically link brittleness and toughness:

*τ* = (*b* + *cB*)/(1 + *aB*) (3)

where: *τ* stands for toughness, *B* stands for brittleness, and *a*, *b*, and *c* are constants.

The formula was developed based on the data obtained for various materials including metals and multiple polymers. Based on their data, authors determined the values of “universal” constants as follows: *a* = −111, *b* = −14,102 and *c* = −1640, with a coefficient of determination *R^2^* = 0.934. The proposed formula can also be expressed as an exponential function, in the following form Equation (4):

*τ* = *dB^e^* (4)

where: *τ*, *B* has the same meaning as in Equation (3) and *d* and *e* are other constant parameters.

The values of *d* and *e* parameters for their data are 178.380 and −0.984, respectively, while for the curve fitted for 3D printed specimens are 183.110 and −1.092, respectively. Moreover, in Figure 5, a data point for ABS according to the calculations of Brostow et al. is presented [27]. It can be seen that 3D printed samples show lower values of toughness and higher brittleness than materials studied by Brostow et al. [27]. Such an effect is often observed during the comparison of 3D printing with conventional processing techniques, such as injection molding. It is associated with the characteristics of the FDM technique, based on layer-by-layer deposition of material, where the following layers are not connected by polymer macromolecules, simultaneously reducing cohesion of specimen [28]. Moreover, as it can be seen in Figure 3 showing a fracture area after tensile tests, 3D printed samples are characterized with significantly improved porosity, which also reduces the mechanical performance of polymeric materials. Nevertheless, it can be seen that the differences between data curves based on literature data and properties of 3D printed samples are not very significant, which suggests that the model developed by Brostow et al. [27] could be considered valid for the analysis of materials obtained with FDM.

Similar to toughness, impact strength determined during Charpy impact tests is a measure of the amount of energy needed to destroy the specimen. The difference between these two parameters is associated with the magnitude of strain rate [29]. Tensile toughness is characterizing materials’ resistance in lower strain rate situations comparing to impact strength. The difference in mechanisms of these two tests is mirrored in the lack of correlation between tensile toughness and impact strength.

Generally, in the case of Charpy impact strength, samples obtained with injection molding are noticeably stronger comparing to 3D printed (additively manufactured samples were able to obtain (36.51%—1W, 44.44%—2W, 39.34%—1B) of injection molding samples), which is the result of significantly more efficient stress transfer mechanisms in the molded sample. Such an effect is related to the fact that, in 3D printed samples, different threads of filament are not bonded together, only deposited next to each other. Therefore, no macromolecular chains are interpenetrating various layers of printed specimens, which facilitates the “delamination” of a sample when stress is applied. Moreover, during injection molding, high pressure is applied to the molten polymer, to precisely fill the mold cavity, which results in a higher density of injection molded samples, comparing to 3D printed ones (1.026–1.033 g/cm^3^ vs. 0.923–0.969 g/cm^3^). Such results implicate the presence of porosity in 3D printed specimens (see Figure 3), which has a noticeable influence on the mechanical performance of prints because, despite using 100% infill, the structure of 3D printed specimen is heterogeneous.

Brostow and Hagg Lobland [30] analyzed the relationship between materials’ brittleness and impact performance since these two properties are closely related to each other. They developed the Equation (5), which is relating Charpy impact strength to material’s brittleness:

*I_c_* = (*a* + 1)/(tanh(*bB*)) (5)

where: *I_c_* stands for Charpy impact strength, *B* stands for brittleness, and *a* and *b* are constants.

**Figure 6:**
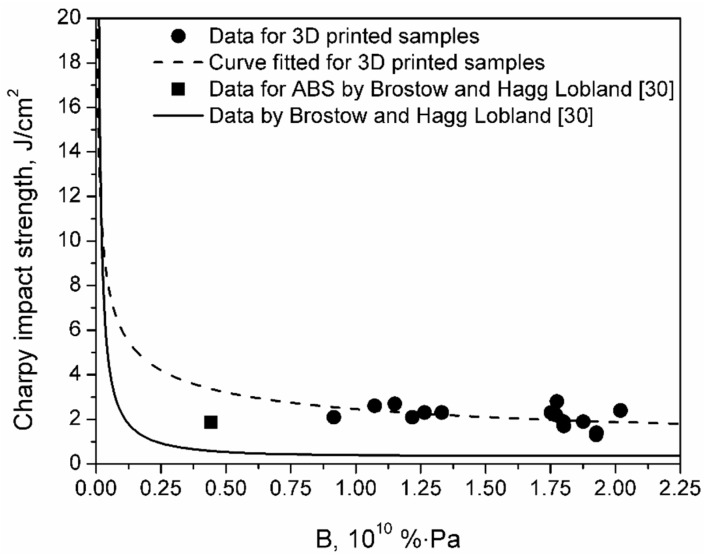
Relationship between Charpy impact strength and brittleness for analyzed materials and comparison with literature data.

For data presented by Brostow and Hagg Lobland [30], values of constants are *a* = −0.640 and *b* = 1.630, and, again, it can be seen that dependence between mechanical properties of 3D printed samples differs from literature data (see Figure 6). However, it can be seen that, surprisingly, data points for investigated specimens lay above the literature curve. It can be concluded that, for a similar level of brittleness, 3D printed samples are characterized with higher than expected impact strength. Such an effect can be associated with characteristics of the 3D printed samples and their morphology. Layer-by-layer deposition of material during printing and proper arrangement of polymer fibers may noticeably enhance the impact performance of the material, which is often used in the designing of laminate structures [31]. As mentioned above, this phenomenon is taken into account during the manufacturing of filament-wound pipes, and just as in the case of these materials, for 3D printed samples, the best impact performance was noted for the fiber arrangement corresponding to 55° winding angle in pipes (see Table 2) [32].

## 4. Conclusions

The presented research paper was aimed at investigation of the impact of raster angle and 3D printing configuration on the static and dynamic mechanical performance of different ABS samples. Performed tests confirmed the noticeable influence of this parameter on the mechanical properties of resulting prints. Moreover, except conventionally investigated raster angles of 45°, 60°, and 90°, we investigated two configurations based on an angle of 55°—first was “conventional”, with orthogonal following layers, while the second was based on optimal winding angle applied during the manufacturing of filament-wound pipes. To the best of our knowledge, such configuration, which provides exceptional properties for pipes, was analyzed in 3D printing for the first time. Although it did not result in enhancement of tensile performance of prints, it provided the highest values of Charpy impact strength for all three types of analyzed ABS materials, despite the highest porosity of the structure exceeding even 10% compared to injection-molded samples. Such results confirm the benefits observed for filament-wound pipes when the analyzed mutual arrangement of the following layers is applied.

Because of the relatively high values of materials’ porosity, resulting from the characteristics of the FDM process and lack of interpenetrating macromolecular chains between particular layers deposited during printing, 3D printed samples showed significant values of brittleness. Comparison with literature data indicated that FDM still cannot be considered as a full-fledged alternative for the injection molding when it comes to the mechanical performance of resulting products. On the other hand, due to some similarities between layer-by-layer deposition of material during FDM printing and manufacturing of laminates, prepared 3D prints showed surprisingly high values of Charpy impact strength, considering their brittleness, overtaking even injection-molded ABS. To sum up, the presented results prove that, despite extensive research works related to the FDM technology, there is still a lot of investigation required for a proper understanding of this process. Further research studies should be more focused on the analysis of overall mechanical performance, not only the most popular tensile and flexural properties. Such an approach would enable tailoring of the performance of final products, which is often emphasized in the case of 3D printing.

## Figures and Tables

**Table 1:** Parameters of the 3D printing process.

Printing Parameter	Value
Nozzle Diameter, mm	0.4
Layer Height, mm	0.1
Fill Pattern	Rectilinear
Fill Percentage, %	100
Perimeters Speed, mm/s	25
External Perimeters Speed, mm/s	12.5
Infill Speed, mm/s	40
First Layer Temperature, °C	240
Other Layers Temperature, °C	230
Bed Temperature, °C	80
Number of Layers	40
Fill Angles, °	45, 55, 55’, 60, 90

**Table 2:** Mechanical properties of 3D printed samples depending on used material and infill angle.

Material	Infill Angle	Porosity, %	Tensile Strength, MPa	Elongation at Break, %	Young’s Modulus, Mpa	Toughness, J/cm^3^	Charpy Impact Strength, J/cm^2^	E’ at 25 °C, Mpa	Brittleness, 10^10^ %·Pa
1W	45	7.48 ± 0.23	37.9 ± 0.2	7.4 ± 1.0	2486 ± 51	196 ± 5	2.1 ± 0.2	1475.1	0.9161
	55	7.58 ± 0.04	37.6 ± 0.4	5.0 ± 0.7	2432 ± 37	139 ± 21	2.3 ± 0.1	1587.6	1.2646
	55’	10.33 ± 0.18	31.9 ± 0.5	4.1 ± 1.7	2112 ± 19	118 ± 19	2.3 ± 0.1	1392.4	1.7517
	60	8.89 ± 0.22	32.0 ± 0.7	3.9 ± 0.5	2196 ± 5	106 ± 11	1.9 ± 0.1	1424.9	1.7995
	90	8.70 ± 0.93	34.4 ± 0.6	3.5 ± 0.6	2336 ± 41	85 ± 8	1.4 ± 0.1	1482.6	1.9271
	IM	-	40.1 ± 0.1	9.6 ± 0.4	2667 ± 42	312 ± 41	6.3 ± 0.6	-	-
2W	45	6.08 ± 0.10	41.7 ± 0.4	5.8 ± 0.2	2348 ± 45	181 ± 7	2.6 ± 0.2	1607.1	1.0728
	55	7.22 ± 0.29	41.2 ± 0.3	5.4 ± 0.5	2327 ± 53	153 ± 7	2.7 ± 0.2	1609.8	1.1504
	55’	9.86 ± 0.98	41.2 ± 0.3	3.4 ± 0.3	2329 ± 64	98 ± 14	2.8 ± 0.1	1657.7	1.7743
	60	7.96 ± 0.40	39.8 ± 0.5	3.5 ± 0.2	2329 ± 68	91 ± 5	2.2 ± 0.2	1613.6	1.7707
	90	8.33 ± 0.31	39.2 ± 0.4	3.6 ± 0.4	2297 ± 57	86 ± 13	1.9 ± 0.4	1480.6	1.8761
	IM	-	48.9 ± 0.6	8.7 ± 0.4	2747 ± 105	318 ± 16	6.3 ± 0.7	-	-
1B	45	6.22 ± 0.13	37.2 ± 0.5	5.7 ± 0.9	2353 ± 50	149 ± 24	2.1 ± 0.2	1440.4	1.2180
	55	6.61 ± 0.34	36.9 ± 0.5	5.2 ± 0.8	2369 ± 37	132 ± 14	2.3 ± 0.1	1444.0	1.3317
	55’	9.80 ± 0.18	33.9 ± 0.3	3.7 ± 0.6	2181 ± 43	96 ± 12	2.4 ± 0.2	1338.7	2.0189
	60	8.45 ± 0.88	35.5 ± 0.5	3.7 ± 0.5	2346 ± 95	91 ± 16	1.7 ± 0.1	1500.1	1.8017
	90	8.11 ± 0.84	33.3 ± 0.7	3.4 ± 0.2	2203 ± 77	79 ± 7	1.3 ± 0.1	1526.9	1.9263
	IM	-	40.9 ± 0.5	9.2 ± 1.7	2544 ± 143	277 ± 31	6.1 ± 0.9	-	-
